# How Are We Managing Patients with Hyperuricemia and Gout: A Cross Sectional Study Assessing Knowledge and Attitudes of Primary Care Physicians?

**DOI:** 10.3390/ijerph18031234

**Published:** 2021-01-30

**Authors:** Sanja Zuzic Furlan, Doris Rusic, Josko Bozic, Mirjana Rumboldt, Zvonko Rumboldt, Marko Rada, Marion Tomicic

**Affiliations:** 1 Department of Family Medicine, University of Split School of Medicine, 21000 Split, Croatia; sanja.zuzic@dz-sdz.hr (S.Z.F.); mirjana.rumboldt@mefst.hr (M.R.); ravnatelj@dz-sdz.hr (M.R.); 2 Department of Family Medicine, Split-Dalmatia County Health Center, 21000 Split, Croatia; 3 Department of Pharmacy, University of Split School of Medicine, 21000 Split, Croatia; doris.rusic@mefst.hr; 4 Department of Pathophysiology, University of Split School of Medicine, 21000 Split, Croatia; josko.bozic@mefst.hr; 5 Department of Internal Medicine, University of Split School of Medicine, 21000 Split, Croatia; zr@mefst.hr

**Keywords:** gout, primary care physicians, knowledge, survey

## Abstract

Background: Studies show that hyperuricemia is an element of the pathophysiology of many conditions. Therefore, the aim of this study was to assess primary care physicians’ knowledge and attitudes toward asymptomatic hyperuricemia and gout management. Methods: A survey-based cross-sectional study was conducted to assess the primary physicians’ attitudes, knowledge, and patient management regarding hyperuricemia and gout. Results: A total of 336 primary care physicians were included. Physicians who read at least one scientific paper covering the topic of hyperuricemia in the past year scored significantly higher in knowledge questions (N = 152, 6.5 ± 2.05 vs. N = 183, 7.04 ± 2.14, *p* = 0.019). Only around half of physicians correctly identified drugs that can lower or elevate serum uric acid levels. Furthermore, the analysis of correct answers to specific questions showed poor understanding of the pathophysiology of hyperuricemia and possible risk factors. Conclusions: This study identified gaps in primary care physicians’ knowledge essential for the adequate management of patients with asymptomatic hyperuricemia and gout. As hyperuricemia and gout are among the fastest rising non-communicable diseases, greater awareness of the available guidelines and more education about the causes and risks of hyperuricemia among primary care physicians may reduce the development of diseases that have hyperuricemia as risk factors.

## 1. Introduction

The final product of purine and protein metabolism in humans is uric acid. Hyperuricemia is defined as an excess of serum uric acid which may lead to the precipitation of uric acid crystals [1,2]. In around 90% of persons with hyperuricemia, there is an insufficient excretion of urate in the kidneys indicating a genetic predisposition [1]. Moreover, serum uric acid levels show a strong heritable component [2]. Other causes include increased endogenous purine production and the consumption of high-purine diets [1].

The prevalence of hyperuricemia is around 20% for both men and women and is increasing, especially in developing countries with a Western lifestyle [1,3,4,5]. A recent study in Poland conducted in persons of 65 years of age and older found that hyperuricemia was present in in 28.2% of women and 24.7% of men [6]. The prevalence of hyperuricemia in Croatia ranges from to roughly from 8.3% to 10.7% (15.4% male, 7.8% female) according to published research [7,8]. Furthermore, research shows a greater risk of hyperuricemia among men compared to women, but for women the risk rises after menopause [2,3,4].

The exact pathophysiology of hyperuricemia is still unclear and is being investigated. However, research indicates that hyperuricemia is closely related to multiple risk factors. Hyperuricemia itself is a risk factor for the development of gout and is the main factor leading to systemic inflammation in gout. Moreover, inflammation in patients with asymptomatic hyperuricemia increases risks for the development of cardiovascular diseases, diabetes, kidney disease and metabolic conditions [1,9].

Studies show that hyperuricemia is an element of the pathophysiology of many conditions. As this list continues to grow, it stresses the need for understanding how well uric acid homeostasis is regulated [2]. It is essential that primary care physicians are familiar with the management of both asymptomatic hyperuricemia and gout, as asymptomatic hyperuricemia poses an increased risk for the development of metabolic and cardiovascular diseases, and gout impairs the quality of life. Cardiovascular diseases are among the leading causes of death in developed countries [10,11]. Moreover, both cardiovascular and metabolic diseases place great strain on healthcare systems [12,13,14]. Therefore, the aim of this study was to assess primary care physicians’ knowledge and attitudes about asymptomatic hyperuricemia and gout management.

## 2. Materials and Methods

### 2.1. Participants

A survey-based cross-sectional study was conducted to assess primary physicians’ attitudes, knowledge, and patient management regarding hyperuricemia and gout. For this purpose, an anonymous survey was distributed by e-mail containing a link to a Surveymonkey^®^ webpage, with the survey used in this study. The e-mail was distributed to the official e-mail addresses of the majority of the eligible primary practices in the country. Furthermore, it was sent to all members of family medicine associations in Croatia via according mailing lists. The study was approved by the Ethics Committee of the Health Centre of the Split-Dalmatia County and the Ethics Committee of the University of Split School of Medicine. Responses were collected during May and June of 2020.

All primary care physicians working on the territory of the Republic of Croatia were considered eligible for inclusion in this study. Participation in the study was voluntary, and physicians received no benefits or compensation for participation. The survey used in the study did not gather data that could be used to reveal the identity of the physician.

### 2.2. Survey

After an extensive literature review that comprised all relevant information regarding knowledge, attitudes and management of hyperuricemia and gout in primary medicine practice, a survey was designed by 3 family physician specialists at the Department of Family Medicine, University of Split School of Medicine. The draft version of the survey consisted of 49 different items, of which 6 items gathered demographic data, 6 items for gout and asymptomatic hyperuricemia management characteristics, while 24 items assessed the physicians’ knowledge, and 13 items were related to attitudes regarding hyperuricemia and gout management. Further evaluation by 2 additional family medicine specialists removed 8 knowledge questions due to the low intelligibility of used phrases, or potentially ambiguous answers. Additionally, a total of 5 attitudes items were removed also due to the low intelligibility of the used phrases, and due to the potential danger of revealing the participants’ identities. The 6 items that gathered demographic data and 6 items about gout and asymptomatic hyperuricemia management characteristics were not changed in any way after this additional assessment. Finally, a 36-item survey divided into 4 parts was agreed upon and distributed among family physicians.

The first part of the survey consisted of 6 items, and it gathered demographic data including gender, age, work experience, qualification, total number of patients in care and population of work location area.

The second part consisted of 6 items as well, and it collected data about the total number of patients with hyperuricemia in care, the average number of patients with asymptomatic hyperuricemia per month, and the average number of patients with gout per month, the percentage of patients with hyperuricemia receiving drug treatment, the referral rate of patients with uric arthritis to a rheumatologist and the number of scientific papers about hyperuricemia read in the past year.

The third part was an evaluation of the participants’ knowledge about hyperuricemia and gout and it consisted of 16 questions related to the management and inter-relation of hyperuricemia and gout. All questions were organized as multiple choice (MCQ), with only one correct answer among 5 offered choices.

The last part consisted of 8 statements that investigated attitudes regarding hyperuricemia and gout management which physicians were asked to rate on a 5-point Likert scale.

The final version of the survey was tested among 18 family physicians for readability, length and understanding of all included items and used phrases. None of the participants reported any difficulties in answering any of the used items.

### 2.3. Statistical Analysis

Sample size analysis was performed with the free Surveymonkey^®^ sample size calculator [15]. The population of family physicians that we investigated in the Republic of Croatia, according to the Croatian Institute for Health Insurance [16], was 2330. With a confidence interval of 95%, and a margin of error of 5%, the needed sample size was 330 family physicians.

Statistical analysis was performed using MedCalc software for Windows (v. 11.5.1.0; MedCalc Software, MedCalc Software Ltd, Mariakerke, Belgium). Results are presented as numbers (proportions) or the mean ± standard deviation where appropriate. The normality of continuous data distribution was tested with the Kolmogorov–Smirnov test. Student’s t-test and one-way analysis of variance were used to determine the differences among physicians’ knowledge scores relative to work experience, number of patients and the number of scientific papers covering the topic of hyperuricemia read in the past year. Finally, a multiple regression analysis adjusted for age and gender was used to assess the independent predictors of the total knowledge score. For this purpose, we used the forward selection algorithm, while unstandardized beta (β) coefficients, standard error (SE), t-value and *p* values were reported. The level of *p* < 0.05 was considered statistically significant.

## 3. Results

This study included a total of 336 primary care physicians (calculated response rate 14.4%), among which there were 275 (81.8%) women. Most of the physicians included in the study were 31–54 years old, and 14% of them were residents. Most physicians had 1500–2000 patients in care and had practices in areas with populations of under 50,000 people (Table 1).

Most physicians stated that they had around 5–10 cases of asymptomatic hyperuricemia per month in daily practice (N = 220, 65.5%), while most stated that they had on average one case of gout per month in daily practice (N = 217, 64.6%). Around 30% of physicians reported that less than 5% of their patients with hyperuricemia received pharmacological treatment. Around half of the physicians included in the study had not read a single scientific paper on asymptomatic hyperuricemia or gout in the past year (N = 152, 45.2%). Moreover, most of them (N = 198, 58.9%) never referred a newly diagnosed patient with uric arthritis to a rheumatologist (Table 2).

Around 60% of physicians believe that guidelines for the management of patients with asymptomatic hyperuricemia would be of great assistance in their everyday practice. Furthermore, physicians (69.6%) greatly valued national referent values of serum uric acid levels as important cut-off margins for making decisions about starting pharmacotherapy in patients with asymptomatic hyperuricemia. More than half of physicians agreed that they are satisfied with their approach regarding their care of patients with asymptomatic hyperuricemia and gout. However, a significant proportion of physicians (42.3%) was not familiar with the European League Against Rheumatism (EULAR) evidence-based recommendations for the management of gout nor do they use them in everyday practice. Most physicians (67.2%) based their approach to patients with asymptomatic hyperuricemia on their clinical experience (Table 3).

**Figure 1:**
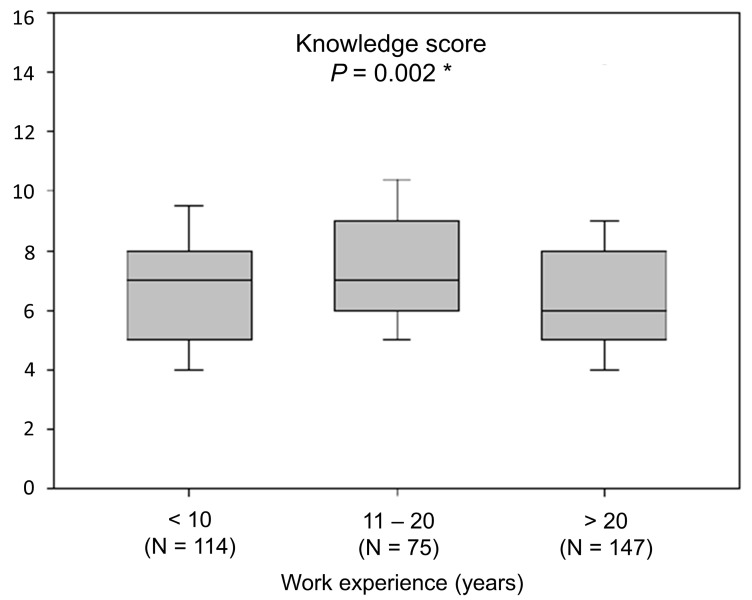
Knowledge score relative to work experience. * Kruskal–Wallis with post-hoc Conover test. Data are presented as median (interquartile range).

Physicians who had read at least one scientific paper covering the topic of hyperuricemia in the past year scored significantly higher in knowledge questions (N = 152, 6.5 ± 2.05 vs. N = 183, 7.04 ± 2.14, *p* = 0.002, range 0–16). Considering work experience, the greatest knowledge scores were found among physicians with 11 to 20 years of work experience (median = 7, IQR 6–9) (Figure 1).

**Figure 2:**
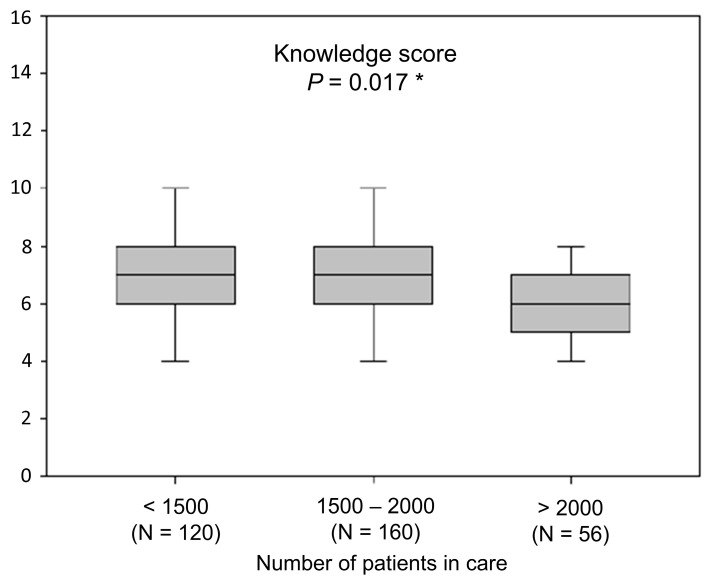
Knowledge score relative to the number of patients in care. * Kruskal–Wallis with post-hoc Conover test. Data are presented as median (interquartile range).

The overall knowledge score of physicians included in the study was 7 (IQR 5–8). Considering the number of patients, the greatest knowledge scores were observed among physicians who had fewer patients (median = 7, IQR 6–8; *p* = 0.017) (Figure 2).

Physicians were unsure about what should be considered asymptomatic hyperuricemia (3%) but were well informed about the non-pharmacological interventions for hyperuricemia and the drugs of choice in the treatment of gout or hyperuricemia. Only around half correctly identified drugs that could lower or elevate serum uric acid levels. Furthermore, the analysis of correct answers to specific questions further showed poor understanding of the pathophysiology of hyperuricemia and possible risk factors (Table 4).

Multiple regression analysis showed that younger physicians with less patients in care were more likely to score higher in knowledge (*p* = 0.002; Table 5).

## 4. Discussion

The results of this study show a modest knowledge of asymptomatic hyperuricemia and gout management among primary care physicians in Croatia. Physicians were most informed about treatment options for asymptomatic hyperuricemia and gout but showed a poor understanding of the underlying pathophysiology and risk factors. In comparison, in a study conducted among primary care physicians in Saudi Arabia, knowledge scores were 3% for mechanisms and 62.7% for dietary recommendations [17]. In the present study, 88.1% knew the non-pharmacological approach to the management of hyperuricemia while 31.1% correctly identified the most common reasons for elevated serum uric acid levels. In a study by Kostka-Jeziorny et al., physicians showed poor awareness of the relationship between hyperuricemia and ischemic heart disease or chronic kidney disease [18].

Hyperuricemia indicates an increased risk of diabetes mellitus or metabolic syndrome and it may lead to hypertension and cardiovascular disease [19,20], therefore it is essential to provide these patients with adequate care. Elevated serum uric acid has been associated not only with several cardiovascular risk factors but also with an increased cardiovascular mortality in the general population [21,22] and after acute myocardial infarction [23,24]. Moreover, in the setting of acute myocardial infarction, hyperuricemia has been linked with an increased inflammatory response [25]. Less than 5% of physicians correctly defined asymptomatic hyperuricemia and were able to identify the goal or main reason for treating hyperuricemia. Our results are consistent with previous research, as studies show that physicians tend to underestimate the effect of hyperuricemia in patients with a high risk of cardiovascular disease [1]. Moreover, a recent study from Poland revealed that a relatively small proportion of physicians are aware of the recommendations for the treatment of hyperuricemia in patients with high cardiovascular risk [18].

A study from Japan showed that only around 10% of patients with hyperuricemia were diagnosed with asymptomatic hyperuricemia in daily practice [3,4]. Most physicians in this study reported having 5–10 cases of asymptomatic hyperuricemia per month in their practice. It is possible that a proportion of cases remains undiagnosed in the primary care setting. Greater understanding of the pathophysiology, possible causes, related conditions and eventual risks of hyperuricemia may raise awareness about the importance of identifying and monitoring patients with hyperuricemia in primary care.

Moreover, the results of this study imply that primary care physicians with fewer patients in care have a greater knowledge of treating these patients. The reason for this is likely a smaller load on these physicians that gives them more time and freedom for education and more thorough patient evaluation. Furthermore, the greatest knowledge scores about of hyperuricemia management were observed among physicians with 11–20 years of work experience. These physicians are young specialists. The results show greater knowledge among physicians who have read at least one scientific paper on this topic in the past year, further stressing the importance of continuous medical education among primary care physicians.

Although most physicians stated that guidelines for the management of patients with asymptomatic hyperuricemia would be of great assistance in their everyday practice, many were also satisfied with their approach to treating patients with asymptomatic hyperuricemia or gout. Considering the modest knowledge among primary care physicians, there is an obvious need for guidelines and education in this area. Not many physicians were aware of EULAR evidence-based recommendations for the management of gout, hence not many used them in everyday practice. These findings are in accordance with previously published research. The lack of education leaves many physicians perceiving gout as an acute disease and offering patients painkillers when necessary rather than long-term medication [26].

Unfortunately, efforts at educating primary care physicians to manage gout effectively and to educate their gout patients sufficiently have not been successful. This issue is aggravated by the fact that gout is mostly managed in primary care and that rates of adherence to urate lowering therapies are 50% or less, worse than most other chronic illnesses [27].

Less than 5% of physicians reported that more than 60% of their patients with hyperuricemia receive pharmacological treatment. A diet with a reduced intake of purine rich foods may reduce urate levels. However, the effects of such an approach are modest and limited, since most patients with hyperuricemia have genetically determined low urate excretion [5,28]. Still, asymptomatic hyperuricemia should generally not be treated, as most of these patients will not develop gout [29]. In the present study, we identified that roughly 88% of physicians are familiar with the non-pharmacological approach to hyperuricemia treatment. However, the results also imply that physicians tend to over-estimate the likely effect of these treatments with only 29.8% providing a correct answer to the question about the effect of non-pharmacological treatment options for lowering hyperuricemia.

In the management of patients with this condition, primary care physicians should be aware of drugs that may raise urate levels [30]. However, in the present study, 62.8% correctly identified medications that may elevate serum uric acid levels. Since patients with hyperuricemia may present with a number of comorbidities [5], it is important that their physicians are aware of the optimal treatment choice available.

### Limitations

This study was survey based and anonymous; however, participants may have provided socially desired answers to some of questions. The survey used in the study was not validated in different populations. This should be taken into consideration when interpreting the results of the study. Additionally, it should be stressed that the data on the number of patients was self-assessed by physicians and was not retrieved from official data records.

## 5. Conclusions

This study identified gaps in primary care physicians’ knowledge essential for the adequate management of patients with asymptomatic hyperuricemia and gout. Primary care physicians are not aware of the risks associated with elevated serum uric acid levels and are in a large proportion unable to correctly identify drugs that may lead to elevated serum uric acid levels. Among physicians in Croatia, not many are familiar with the EULAR guidelines but think that they would benefit from guidelines for the management of such patients. As hyperuricemia and gout are among the fastest rising non-communicable conditions, greater awareness about the available guidelines and more education about the causes and risks of hyperuricemia among primary care physicians may contribute to a reduction in the development of other diseases that have hyperuricemia in their background. In the present study, we identified that 62.8% of physicians correctly identified drugs that elevate serum uric acid levels and 47% correctly identified drugs that lower serum uric acid levels. In addition, the results implicate that physicians are unable to provide an accurate estimate of the effect of non-pharmacological interventions for the treatment of hyperuricemia and gout. For future studies it would be of great interest to see the percentage of physicians who would prescribe the different therapeutic options (urate lowering therapy, non-steroidal anti-inflammatory drugs, colchicine, corticosteroids) for patients with asymptomatic hyperuricemia considering patient’s comorbidities as well as to further investigate knowledge about the effect of diet and other non-pharmacological interventions in patients with hyperuricemia.

## Figures and Tables

**Table 1:** Demographic characteristics of study participants.

Total	336 (100%)
Gender (N)	
Men	61 (18.2%)
Women	275 (81.8%)
Age (years)	
<30	46 (13.7%)
31–54	171 (50.9%)
>55	119 (35.4%)
Work experience (years)	
<5	55 (16.4%)
5–10	59 (17.6%)
11–20	75 (22.3%)
>20	147 (43.7%)
General practitioner	106 (31.5%)
Family medicine specialist	183 (54.5%)
Family medicine resident	47 (14.0%)
Number of patients in care	
<500	5 (1.5%)
500–1000	25 (7.4%)
1000–1500	90 (26.8%)
1500–2000	160 (47.6%)
>2000	56 (16.7%)
Population of work location area	
<5000	103 (30.7%)
5000–50,000	88 (26.2%)
50,000–150,000	36 (10.7%)
150,000–300,000	51 (15.2%)
>300,000	58 (17.3%)

Data are presented as whole numbers (proportions).

**Table 2:** Gout and asymptomatic hyperuricemia management practices.

Patients with Hyperuricemia in Care (N)	
<100	227 (67.6%)
100–300	94 (28.0%)
>300	15 (4.5%)
Cases of asymptomatic hyperuricemia in daily practice (N per month)	
0	8 (2.4%)
1	61 (18.2%)
5–10	220 (65.5%)
11–29	36 (10.7%)
≥30	11 (3.3%)
Cases of gout in daily practice (N per month)	
0	32 (9.5%)
1	217 (64.6%)
5–10	83 (24.7%)
11–29	4 (1.2%)
≥30	0 (0.0%)
Patients with hyperuricemia receiving pharmacological treatment in care	
≤5%	101 (30.1%)
6%–10%	73 (21.7%)
11%–30%	95 (28.3%)
31%–60%	51 (15.2%)
>60%	16 (4.8%)
Scientific papers on asymptomatic hyperuricemia/gout read in the past year (N)	
0	152 (45.2%)
1	126 (37.5%)
2	40 (11.9%)
3	9 (2.7%)
4 and more	9 (2.7%)
How often do you refer a newly diagnosed gout patient to a rheumatologist?	
Never	198 (58.9%)
In 10% of cases	119 (35.4%)
In 11%–30% of cases	8 (2.4%)
In 31%–50% of cases	6 (1.8%)
Consistently	5 (1.8%)

Data are presented as whole numbers (proportions).

**Table 3:** Attitudes about gout and asymptomatic hyperuricemia treatment.

	Fully Disagree	Disagree	Unsure	Agree	Fully Agree
I am satisfied with my approach to care of patients with asymptomatic hyperuricemia.	2 (0.6%)	41 (12.2%)	128 (38.1%)	147 (43.7%)	18 (5.4%)
I am satisfied with my approach to care of patients with gout.	1 (0.3%)	20 (6.0%)	83 (24.7%)	199 (59.2%)	33 (9.8%)
I am satisfied with my success in changing lifestyle of patients with asymptomatic hyperuricemia/gout.	25 (7.4%)	110 (32.7%)	123 (36.6%)	74 (22.0%)	4 (1.2%)
I am familiar with the EULAR evidence-based recommendations for the management of gout.	59 (17.6%)	83 (24.7%)	102 (30.4%)	81 (24.1%)	11 (3.3%)
I use EULAR evidence-based recommendations for the management of gout in everyday practice.	66 (19.6%)	76 (22.6%)	112 (33.3%)	78 (23.2%)	4 (1.2%)
I approach patients with asymptomatic hyperuricemia mostly based on my clinical experience.	8 (2.4%)	28 (8.3%)	74 (22.0%)	194 (57.7%)	32 (9.5%)
I believe that guidelines for management of patients with asymptomatic hyperuricemia would be of great assistance in my everyday practice.	2 (0.6%)	3 (0.9%)	26 (7.7%)	105 (31.2%)	200 (59.5%)
National referent values of serum uric acid levels are important cut-off values for everyday decisions about starting pharmacotherapy in patients with asymptomatic hyperuricemia.	2 (0.6%)	20 (6.0%)	80 (23.8%)	148 (44.0%)	86 (25.6%)

Data are presented as whole numbers (proportions); EULAR-European League Against Rheumatism

**Table 4:** Physicians who answered correctly to questions related to asymptomatic hyperuricemia and gout.

	Number (%) of Physicians Who Answered Correctly
1. Non-pharmacological interventions for hyperuricemia	296 (88.1%)
2. Drug classes for treatment of hyperuricemia registered in Croatia	261 (77.7%)
3. Treatment for gout flares	260 (77.4%)
4. Second line of treatment of hyperuricemia	250 (74.4%)
5. Treatment approach in asymptomatic hyperuricemia	233 (69.3%)
6. Identifying drugs that elevate serum uric acid levels	211 (62.8%)
7. Identifying drugs that lower serum uric acid levels	158 (47.0%)
8. Diagnostic procedure for confirmation of gout diagnosis	122 (36.3%)
9. Relationship of asymptomatic hyperuricemia and gout	115 (34.2%)
10. The expected effect of non-pharmacological treatment options for lowering hyperuricemia	100 (29.8%)
11. Drugs for lowering hyperuricemia registered in Croatia (with reference to the most likely cause of hyperuricemia in most patients)	94 (28.0%)
12. Cut-off value of serum uric levels for initiation of pharmacological treatment	71 (21.1%)
13. Asymptomatic hyperuricemia as a risk factor	46 (13.7%)
14. Most common reason of elevated urate levels	44 (13.1%)
15. The goal when treating hyperuricemia	13 (3.9%)
16. Definition of asymptomatic hyperuricemia	10 (3%)

Data are presented as whole numbers (proportions).

**Table 5:** Factors influencing knowledge about asymptomatic hyperuricemia and gout.

Factor	Β	SE	t	*p* *
Age (years)	−0.667	0.224	−2.983	0.003
Work experience (clustered)	0.133	0.172	0.772	0.440
Number of patients in care (clustered)	−0.293	0.129	−2.261	0.024
Gender	0.198	0.293	0.499	0.499

* multiple regression analysis; SE—standard error.

## Data Availability

The data presented in this study are available on request from the corresponding author. The data are not publicly available due to ethical restrictions.
